# Bw4 ligand and direct T-cell receptor binding induced selection on HLA A and B alleles

**DOI:** 10.3389/fimmu.2023.1236080

**Published:** 2023-11-21

**Authors:** Reut Levi, Lee Levi, Yoram Louzoun

**Affiliations:** Department of Mathematics, Bar-Ilan University, Ramat Gan, Israel

**Keywords:** selection, HLA, balancing, machine learning, allele, Bw4, T cell receptor

## Abstract

**Introduction:**

The HLA region is the hallmark of balancing selection, argued to be driven by the pressure to present a wide variety of viral epitopes. As such selection on the peptide-binding positions has been proposed to drive HLA population genetics. MHC molecules also directly binds to the T-Cell Receptor and killer cell immunoglobulin-like receptors (KIR).

**Methods:**

We here combine the HLA allele frequencies in over six-million Hematopoietic Stem Cells (HSC) donors with a novel machine-learning-based method to predict allele frequency.

**Results:**

We show for the first time that allele frequency can be predicted from their sequences. This prediction yields a natural measure for selection. The strongest selection is affecting KIR binding regions, followed by the peptide-binding cleft. The selection from the direct interaction with the KIR and TCR is centered on positively charged residues (mainly Arginine), and some positions in the peptide-binding cleft are not associated with the allele frequency, especially Tyrosine residues.

**Discussion:**

These results suggest that the balancing selection for peptide presentation is combined with a positive selection for KIR and TCR binding.

## 1 Introduction

A major challenge in understanding the evolutionary forces that act on species and affect their genetic variation is the identification of loci and positions under selection. In a simple model of directional selection, a novel mutation is favored if it confers a selective advantage to the organism (positive selection) (1). However, in some loci, balancing selection has been proposed to favor a large number of alleles in the same locus (2).

A hallmark of balancing selection is the MHC (See **Table 1** for all abbreviations) region, encoding the MHC molecule that presents peptides to T lymphocytes (3), denoted HLA in humans. The HLA region is the most diverse loci in the human genome (4). The selection has been argued to emerge from the need to bind peptides from different pathogens. As such, it is centered on peptide-binding positions in the MHC molecule (5). Classical HLA genes include two main groups - A, B and C denoted class I presenting intra-cellular peptides, and DR and DQ denoted class II, typically presenting extracellular peptides. Most of the variations among alleles are indeed concentrated in the peptide-binding regions in the second and third exons of the class I loci and the second exon of the class II loci (6). We currently have limited accuracy of DP allele frequencies. Thus, DP was not studied in the current analysis.

**Table 1:** List of acronyms used in the current analysis.

AA	Amino Acid
TCR	T-Cell Receptor
MHC	Major Histocompatibility Complex
HLA	Human Leukocyte Antigen
CDR	Complementarity-Determining Region
KIR	Killer cell Immunoglobulin-like Receptor
NK	Natural Killer
PB	Peptide-Binding
NPB	Non-Peptide-Binding
LILR	Leukocyte Immunoglobulin-Like Receptor
TSP	Trans-Species Polymorphism
ESP	Electrostatic Surface Potential
SVR	Support Vector Regression
RBF	Radial Basis Function

The main evidence for balancing selection in HLA are trans-species polymorphism (TSP) and high diversity. Many distinct mechanisms have been proposed to induce this balancing selection (7), including direct selection by pathogens, heterozygote advantage (8, 9), MHC-dependent mate choice (assortative mating) and sexual selection, including MHC dependence on mother-fetal interactions and the apparent olfactory recognition of MHC haplotypes (10).

However, in humans, MHC-I also has direct interactions with three other molecules that could affect the selection of HLA alleles. The MHC-I molecule has direct interaction with the TCR and plays a role in TCR-HLA peptide binding. Recently, the direct interaction of the TCR and the HLA was shown to be affected by the V gene and CDR3 sequence of the TCR *β* chain (11–14). NK cells also bind MHC-I molecules via two distinct groups of receptors, killer immuno-globulin-like receptors (KIRs) and CD94:NKG2. Natural killer cells are lymphocytes of the innate immune response that provide an important defense against infection, particularly viral infections (15–17). KIRs are inhibitory and activating receptors expressed mostly on the surface of NK cells and some T-cells. KIRs recognize broad groups of HLA class I molecules, mainly through the Bw4 binding domain in the A and B HLA alleles (18). Bw4 is a public epitope present on a subset of HLA-B and on some HLA-A alleles. NK cells can induce cell death in cells lacking Bw4.

MHC-I molecules are also the ligands for the leukocyte immunoglobulin-like receptors (LILR) of which LILRB1 and LILRB2 are the best characterized (18). A variety of HLA allotypes bind LILRB1 and LILRB2 with varying affinities, especially LILRB2, which shows considerable variation across HLA alleles (19). The LILRB1 and LILRB2 receptors are inhibitory receptors found mainly on myeloid cells such as dendritic cells and macrophages; signaling via LILR influences their activation (20). We here show that in humans, the direct interaction of MHC-I with TCRs and KIR molecules has a direct signature of selection in the HLA region.

Several methods were proposed for the identification of positions associated with selection (21), including among others, the examination of surplus in heterozygous genotypes (22), identification of local uplifted genetic variance (23), polymorphisms (24), changes in the range of sites frequencies toward common frequencies (25–27), deviation of genetic diversity from neutral models (28), presence of trans-species polymorphism (29, 30), explicit models of polymorphism patterns (31, 32), correlation of environmental features and allele frequencies (33), and others. Most of these methods are based on the distribution of nucleotides and amino acids at the appropriate position. As such, they are indirect evidence for selection. Recently some frequency-based methods were also developed (34–37). Such methods are based on the principle that non-neutral evolution leaves a signature of selection on the allele frequencies.

A more direct measure of selection would be to measure the effect of each amino acid in each position on the allele frequency. While in most genes the sampling depth and the polymorphism do not allow for such a direct measurement, the HLA locus is polymorphic enough (over 24,000 alleles in A, B, and C, and more than 7,000 as defined by the amino acid sequence of exons 2 and 3 only), and has a large enough coverage (over 39 million typed donors worldwide) (38, 39). We have recently demonstrated the validity of the frequency estimates of HLA haplotypes and their adequacy for population structure modeling (37, 40, 41). We here show for the first time that allele sequence can be used to predict allele frequency on a test set. We then show that the coefficients of the prediction algorithm highlight a strong additional selection induced on the HLA locus on regions not binding the peptide, but rather NK cells or directly the T-Cell Receptor (TCR). To the best of our knowledge, this is the first prediction of allele frequency in the human population from their sequence in any locus.

## 2 Results

**Figure 1:**
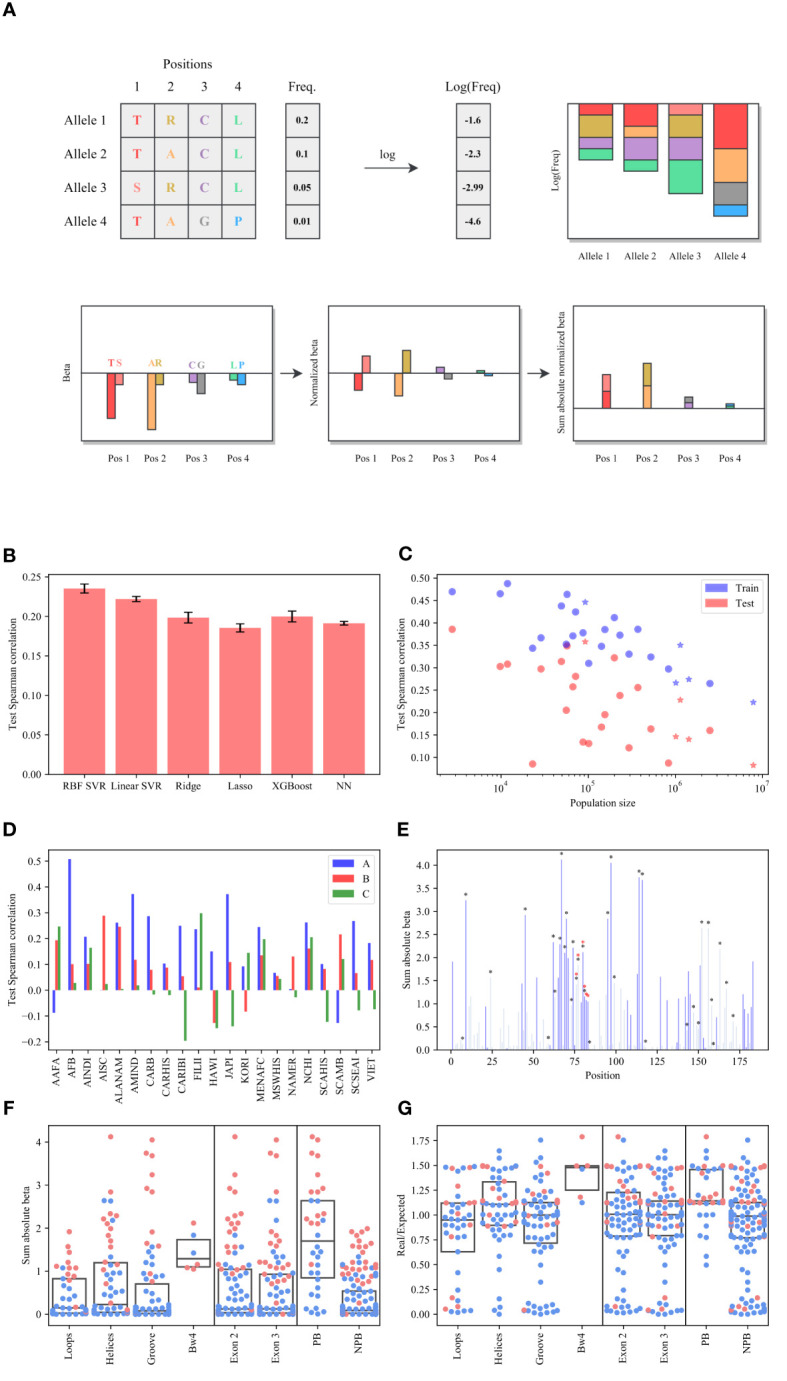
Frequency prediction. **(A)** Schematic description of $S_{\beta }$ estimate. We regress the log frequency on the AA in all positions and obtain a coefficient β per AA per position. We normalize the sum of β to be 0 in each position. $S_{\beta }$ is the sum of the absolute of β values per position. **(B)** The average Spearman correlation test between the predicted and real log frequencies over all populations for different models. Complex models actually have a worse prediction than linear models. **(C)** Spearman correlation as a function of the population. The stars represent the broad populations (AFA, API, CAU, HIS and NAM). **(D)** The Spearman correlation between the log real y values (allele’s frequencies) and the predicted ones by SVR on the test set (the amino acid sequence of each allele) for each locus separately and each population, where the blue bars represent the A locus, the pink bars represent the B locus and the green bars represent the C locus. The A and B loci have consistent positive correlations, while the C locus has no correlation. **(E)** The sum of the absolute of β values per position (the β values are defined as the regression coefficient of the SVR), where the black/redstars represent the peptide-binding or the Bw4 positions respectively, and the dark blue bars represent the significant positions. **(F, G)** The distribution of the $S_{\beta }$ values **(F)** and the $d_{n}/d_{s}$ values **(G)** for each region.Pink dots are significantly different from the null model.


To show that the HLA allele amino acid composition can be used to predict the frequency of an unseen allele, we regressed the log allele frequency on the amino acid composition represented as a one-hot per position (see Online Methods for formalism and training-test division and **Figure 1A** for a schematic scheme). We used the HLA allele frequencies imputed from the HLA typings from 6.59 million donors of the National Marrow Donor Program registry. The frequencies are divided into 21 detailed and 5 broad sub-population across the US (42) (see **Supp. Mat. Table S1** for details). We tested multiple linear and non-linear regression methods for each population. Formally, for the linear regressors, each population j , and each allele i in locus L (A, B or C), with frequency $a_{i,j}$ , we mark:

(1) $$loss=\sum^{}{\left(log_{10}\left(a_{i,j}\right)-\sum_{k}\beta_{j,k}*x_{i,k}\right)}^{2}+g\left(\beta \right),$$

where $x_{i,k}$ is a one-hot representation of the allele sequence, $\beta_{j,k}$ are the coefficients for the appropriate population and g(β) is a regularization term that varies among methods (e.g. Ridge LASSO). The formalism is similar in non-linear methods (see Methods). We have also tested the possibility of regression of all loci simultaneously. In such cases, an additional term was added to the regression representing the locus. Finally, we also performed a similar regression on all populations simultaneously. In this case, an additional one-hot term was added for the population (see Methods).

The RBF Support-Vector Regression (SVR) produced the highest average test correlation (**Figure 1B**), but a linear SVR had almost similar scores (ANOVA test between all the models p<9.74e−27 , T-test between the RBF SVR and the linear SVR p<0.001 ). Thus, to get a simple explanation of the coefficients, we trained the SVR model with the linear kernel on all loci together. We thus used the linear SVR model for all loci together.

Note that more precise results can be obtained for specific loci and populations using other models (**Supp. Mat. Table S9**), but as further mentioned their coefficients fail to detect previously reported selection, and were thus not used. Moreover, the samemodel is mainly predicting the difference between populations, and not the direct effect of the sequence on the log-frequency.

The correlation between the predicted and real log frequencies decreases with the population size (**Figure 1C**, the broad populations (AFA, API, CAU, HIS and NAM) are marked with a star), as a result, the correlation for the broad groups is lower than for the detailed groups in general. We thus focus on the detailed groups in the remainder of the analysis. The correlation is highest in the A locus (**Figure 1D**), followed by B (0.178 vs 0.102). The average correlation in the C locus is almost null (0.03) and non-significant.

This may be due to the sequence differences and failure to learn from A and B to C which has fewer alleles. To check that this is not the case, we performed a regression on each of the loci separately. Again, in the C locus, the prediction models fail to predict the frequencies of the alleles (**Supp. Mat. Figure S3**). Therefore, the lack of prediction at the C locus is not due to its difference from the A and B loci, nor is it because of the number of alleles, which is similar among loci (2,196 in C vs 2,477 in A and 3,219 in B).

In the linear models, each amino acid at each position is associated with a coefficient ( β ), we computed the sum of the absolute of β values ( $S_{\beta }$ ) per position. A low $S_{\beta }$ implies that mutations in this position have a minimal effect on the allele frequencies, and a high $S_{\beta }$ implies that some AAs in this position are strongly correlated with a high or low allele frequency. As is the case for most selection measures, this is no proof of causality, since different positions may be in Linkage Disequilibrium (LD).

The regression coefficients were consistent among the different populations, with an average correlation of 0.3±0.009 over the large enough coefficients ( $\sum_{i}|\beta_{i,j,k})|>10^{-2}$ , when computing the correlation on all positions, it is closeto 1, but this is because many positions have values near 0).

$\;S_{\beta }$ is per definition biased toward positions with a more diverse amino acid composition (**Supp. Mat. Figure S4**). This is expected since such positions are also the ones most associated with selection. Still, we have examined several possible methods of $S_{\beta }$ estimation, including the sum of absolute values (as above), the average of absolute values, and the average of absolute values weighted by the frequency of each AA at the appropriate position. The sum of absolute values best reproduces known results on the selection affecting the peptide binding domain and was thus kept (**Supp. Mat. Figure S5A** vs **S5B, C**). Similarly, a single model trained on all the populations together had less distinctive $S_{\beta }$ values in the peptide binding region than outside (**Supp. Mat. Figure S5D**), and was thus ignored.

As expected, the positions with the highest $S_{\beta }$ are the peptide-binding region positions. However, surprisingly, those are followed by the Bw4 KIR ligand (see Online Methods for the definition of HLA positions, **Figure 1E** and **Supp. Mat. Figure S8**), where the black/red stars represent the peptide-binding or the Bw4 positions respectively). The dark blue bars represent the significant positions. A significant position is defined as $S_{\beta }$ larger than the 95th percentile of the $S_{\beta }$ values in the null model (where all the frequencies are mixed - see Methods). Note that there is some overlap between PB and Bw4. However, there is a clear selection for Bw4. Positions 80-83 are significantly selected, and only 80,81 are PB, while 76 and 77 are not selected and are PB.

We further divided the 183 positions of exons 2 and 3 into 4 regions (the loop, helices, groove, and the Bw4 region), two exons (exon 2 and exon 3 regions) and peptide-binding/non-peptide-binding regions (PB vs NPB - see **Table 1** for all abbreviations and **Supp. Mat. Table S5** for all groups’ positions). We computed $S_{\beta }$ for each region (**Figure 1F**). The $S_{\beta }$ values in the Bw4 region and the PB region are the highest, and significantly different than others for A and Bloci. No difference was detected between the other divisions (Kruskal Wallis test and U-test p-values results are shown in **Supp. Mat. Table S4**).

To validate the selection in positions outside the peptide binding domain, we compared the $S_{\beta }$ based prediction to a more classical (albeit indirect) method - the ratio of non-synonymous to synonymous substitutions ( $\omega =d_{n}/d_{s}$ ) 43 population. ω measures selection pressures by comparing the rate of synonymous ( $d_{S}$ ) and non-synonymous substitutions ( $d_{N}$ ) at each codon. The expected ratio $d_{N}/d_{S}$ is computed assuming an equal mutation rate at all positions, but different rates between or within purines and pyrimidines. If selection favors new mutations affecting the phenotypes, a higher ratio is expected, and vice versa. This intuitive interpretation of $d_{N}/d_{S}$ is supported by theoretical work on the relationship between the $d_{N}/d_{S}$ statistic and the underlying selection pressure in a Wright-Fisher model (44, 45).We compared the $S_{\beta }$ based results with the ω based results and obtained a similar trend, but a much clearer signal of $S_{\beta }$ (**Figure 1F**) than for $d_{n}/d_{s}$ (**Figure 1G** for all loci and **Supp. Mat. Figure S1** for A, B, and C separated) in Bw4 and PB. Note that many NPB positions also have high and significant $S_{\beta }$ values and some PB have low $S_{\beta }$ values, as further discussed. We have repeated the results here with a Kimura model (46), with similar results (**Supp. Mat. Figure S6**).

High $S_{\beta }$ values may simply represent the appearance time during HLA evolution. AA appearing at some position early in the HLA evolution can be expected to be associated with frequent alleles. To test if high β values only represent evolution time, the time of appearance of each amino acid in each position in the HLA phylogeny was compared with $S_{\beta }$ .

**Figure 2:**
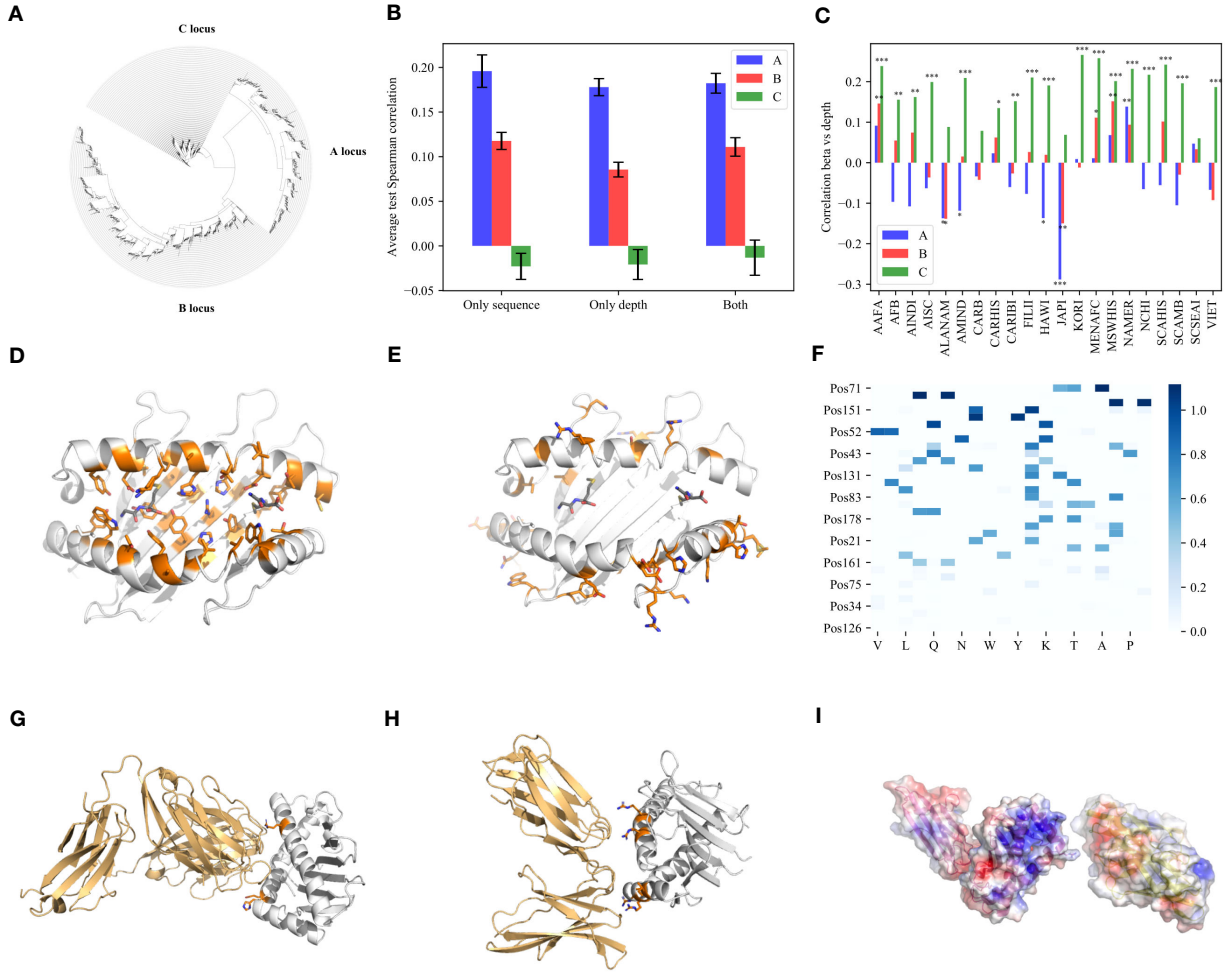
Depth. **(A)** A phylogenetic tree built with all the HLA class I nucleotide sequences, using RDP4 and the maximum likelihood algorithm on A, B and C loci together. Except for very rare cases, A, B and C are clustered separately. **(B)** The average test correlation over all populations for each of the three models (only sequence, only depth and both). The blue bars represent the A locus, the pink bars represent the B locus and the green bars represent the C locus. **(C)** The correlation between the weights ( β ) and depth ( H ) vectors over all positions and AA for each population separately. **(D, E)** PyMOL visualization of the positions side chain in the PB region **(D)** and in the significant NPB region **(E)**. **(F)** Heatmap of β(AA,Pos) values of all the significant positions in the NPB region. One can clearly see a dominant effect of Arginine (R). **(G, H)** PyMOL visualization of the MHC class I and the positions in the significant NPB region binding to TCR **(G)** and KIR molecules **(H)**. The orange color represents the positions and their side chain. **(I)** PyMOL visualization for the electrostatic surface potential (ESP) of MHC (left) and KIR (right). The blue color represents positive EPS and the red color represents negative EPS.

The HLA locus is known to have passed recombination and gene conversion events (47). Thus, standard phylogeny may fail to capture the HLA locus evolution. To detect such events, we first built a tree of all class I alleles together (**Figure 2A**), using RDP4 (48). RDP4 builds phylogenies and in parallel, detects recombination events. When the phylogeny of A, B, and C HLA alleles was computed on the same tree, a clear separation into the A, B, and C loci appears, except for B*07:13, B*67:02, B*73:01, and B*73:02, which appeared on different branches of the tree than the other alleles in their locus (**Figure 2A**, for a higher resolution view of the tree, please refer to the following link: https://itol.embl.de/tree/109672384336311547023541). We repeated the analysis per locus (A, B, and C) without these alleles to detect within locus recombination events, which were further removed from the analysis (37 out of 7,892 alleles to have passed recombinations or gene conversion within exon 2 or 3 - **Supp. Mat. Table S2**). We thenanalyzed exons 2 and 3 separately to avoid between exon recombinations (49) using the PHYLIP package (50), without the removed alleles mentioned in **Supp. Mat. Table S3**. Some of these events were previously reported and others are new. The phylogeny was performed at the amino acid level to be consistent with the regression analysis. Thus, amino acid conserving convergent evolution events (the same amino acid with different nucleotides (51)) were ignored.

We defined H(AA,Pos) to be the average depth of each AA in the phylogenic tree of all HLA alleles (see Online Methods) at each position. We then tested whether the allele frequencies can be predicted using H(AA,Pos) . Three models were tested: A) Only sequence The sequence model used above. B) **Only depth** prediction using only H(AA,Pos) , and 0 when an AA was observed less than 3 times in a position, as in the first model. C) **Both** Both values as input (**Figure 2B**). Note that the depth model contains the sequence information since it also has 0 for AA not in the sequence, but it also contains information of the depth of each AA. One can see adding depth does not improve the prediction accuracy. Thus, the allele frequency is not strongly affected by the appearance time of its amino acids if at all. To further test for association between β(AA,Pos) and H(AA,Pos) , we computed the correlation between the weights in the depth independent model (A) and the depth vectors: β vs H over all positions and AA for each population separately (**Figure 2C**). In the A and B loci, correlations are weak and around 0, while in the C locus, correlations tend to be positive and very significant ( p<0.001 ) (**Figure 2C**). Thus, the allele frequency in the C locus is strongly associated with their appearance time, in contrast with the A and B loci (**Figure 2B**). The time of appearance is not generalizable to new alleles. As such it cannot be used to predict frequency. This is consistent with the lack of prediction of the C allele frequencies using β . Note that if allele frequency would be fully driven by peptide-binding, one would expect no difference between A, B and C.

Beyond the selection induced by Bw4 and PB domain, there are positions in the PB region with low $S_{\beta }$ value, and positions not in PB and not in Bw4 with high beta value. Out of the 9 insignificant positions in the PB region with $S_{\beta }<1$ , 6 of them (66%) are Tyrosine (**Supp. Mat. Table S7** for all AAs of these positions), Tyrosine is known for its low evolution rate, among others, because of the neighboring stop codon (52).

In contrast, there are also high $S_{\beta }$ values in the NPB region (**Supp. Mat. Table S6**). We used PyMOL (53) to compute the positions of their side chain. Interestingly, all these positions are predicted to face outside of the binding cleft toward the T-cell itself or other binding cells (**Figure 2E**), in contrast to the positions in the PB region that face the binding cleft (**Figure 2D**), suggesting a selection mediated by the direct interaction with other cells rather than the peptide.

There are two natural candidates for inducing this selection - T-cells and NK cells. To compare those, we used 3 TCR-MHC-I structures and 3 MHC-I KIR interactions with 3DL1, 2DL1 and 2DL2 receptors. We then computed the positions on the MHC molecule closest to the KIR or the TCR. 4 out of 34 significant positions were found to directly bind the TCR (positions 65, 151, 154 and 161) (**Figure 2G** and **Supp. Mat. Figures S2A, B**). 6 out of the 34 were computed by PyMOL to directly bind KIR molecules (positions 151, 145 and 79 were found to be common among all the structures, but in addition positions 142, 75 and 83 were also found in specific structures) (**Figure 2H** and **Supp. Mat. Figures S2C, D**). Most of these positions are Arginines. Some of those were found to bind two different KIR receptors. These results suggest a strong charge-mediated effect of KIR binding positions beyond the Bw4 domains, not only in B, but also in A HLA alleles. Note that the TCR variability is large. Thus, the three tested TCRs here may not represent the full variability, and the significant SPB that point outside may bind different TCRs.

To further understand the possible effect of charge on the difference in $S_{\beta }$ among HLA positions with side chains toward other binding cells, we analyzed the β(AA,Pos) values of all the significant positions in the NPB region (**Figure 2F**). We performed a Chi-Square test between the sum of the $S_{\beta }$ values for each amino acid and the sum of the $S_{\beta }$ values when mixing all these values (see Online Methods). The top 4 AA are R, G, H and K, with R the most significant, suggesting again that selection is strongly associated with a positive charge. A selection for charge may be simply the result of an opposite charge on the 2DL1 binding site. Indeed, when computing the electrostatic surface potential of 2DL1 molecules in front of the positions computed to bind 3DL1 in the MHC a clear negative chargecan be observed (**Figure 2I**, and detailed view in **Supp. Mat. Figure S7**). Note that a positive charge was previously reported to be crucial in Immunoglobulin binding, especially in the context of autoimmunity (54, 55). We here suggest that selection for positive charge in binding TCR and KIR may also be crucial.

## 3 Discussion

Most population genetics methods use indirect measures to explain the gene diversity in present populations and the allele and genotype frequencies and identify selection pressures. We have here analyzed the Human Leukocyte Antigen (HLA) genes and shown that the sequence of HLA A and B alleles can be used to predict the appropriate alleles log frequency with a linear model, where each amino acid at each position contributes a constant value to the allele log frequency. The linear model has been found to be much better than the tested non-linear model suggesting that epistatic effects are limited. Interestingly, the relation between AA sequence and frequency was only present in A and B alleles suggesting a mechanism beyond peptide binding, which is similarin A, B, and C loci.

The relation between AA at a given position and the allele frequency can be explained by either selection or the time since the AA’s first appearance in the phylogeny. An AA can be associated with a large allele frequency, either because it contributes to the fitness of the phenotype, or because it is ancient. We have previously addressed this problem through the branch imbalance following mutations (36). Given the very large number of alleles with measured frequencies, we could here compare directly the depth of each AA with its contribution to the allele frequency. We have shown that in the C locus, depth and contribution to size are highly correlated, but not in A and B.

To measure selection, we defined a novel score $S_{\beta }$ for the relation between sequence and frequency, based on the sum of the regression coefficients’ absolute values. Applying this score to the MHC class I shows a clear selection in PB positions. However, there were many significant positions in the NPB region, with the strongest selection occurring at the Bw4 ligand. We computed the orientation of the AA side chains and showed that many of them bind directly to KIR even beyond the Bw4 regions. Some of the remaining positions bind directly to the TCR. We found no evidence for selection in LILR binding positions. While there are some sources in the literature of HLA positions that are reported to be bound to the LILR receptor (18), the current analysis was limited to exon 2 and 3, and the LILR binding region being farther away from the peptide binding cleft may affect other loci.

HLA allele frequencies have been argued to be mainly selected by a balancing selection for peptide-binding (56). However, our recent results suggest that the selection affecting the HLA region may be much more complex and dominated by a purifying selection at the haplotype level (37, 41). We have here shown at the AA level that a very strong selection is induced by charge-mediated interactions between KIR and TCR and the MHC molecules. Such a selection may favor specific haplotypes in parallel with the binding peptide-induced balancing selection on alleles.

Multiple caveats have to be considered when analyzing these results. The most significant is the known Linkage Disequilibrium (LD) between HLA genes (57). Selection in the HLA locus may not limited to single genes, but may work on full haplotypes. Thus, the frequency of a gene in a population may actually be affected by other genes. This may explain the limited accuracy of the prediction based only on each gene sequence. A combined haplotype-based score may improve the accuracy of the current predictor and will be further studied. Another important caveat is the effect of AA diversity. The current selection score is affected by the number of AA candidates in each position. We have tested different score combinations. A score that would avoid this dependence may further improve the accuracy of the selection estimation.

An interesting conclusion from the current study would be that some new alleles may have a higher probability of emerging in the population. To predict such alleles, one would need beyond the current results, a model for the generation probability of alleles from the existing ones.

## 4 Methods

### 4.1 Data

For the lineage analysis, we used the HLA class I allele’s exon 2 and exon 3 sequences from the IMGT/HLA Database (58). To compute the allele’s frequencies, we used the data of 6.59 million donor HLA typing from the National Marrow Donor Program Registry (37, 42, 59). It consists of the abundances of all different HLA haplotypes in the registry. Allele frequencies were derived as marginal sums of the haplotype frequencies. For example, to compute the one-locus A frequencies for a given allele, we merged all extended A C B DRB1 DQB1 haplotypes with the appropriate A allele into an A allele frequency (42).

### 4.2 Training test split

We divided the data into training and test sets using the *train_test_split* method from the python scikit-learn library (60). The first group constitutes 80% of the data and was used for training and finding the best hyperparameters. The second group constitutes 20% of the data and was used as an external test. All the results are reported on the test group.

### 4.3 Neural Network Intelligence

NNI (61) was used for parameter hypertuning. For each algorithm, NNI was used in two steps for a broad hyperparameter tuning. First, a grid search of a wide range of parameters was performed to get the amplitude of the regularization. The second step was to refine the outcome by setting the tuner to Tree-structure Paezen Estimator (TPE) and running another search, while considering historical measurements. We then found the hyperparameters that produce the highest Spearman correlation on the internal validation set (our metric). The search space of the hyperparameters for the best model, SVR, is presented in **Supp. Mat. Table S8**.

### 4.4 Prediction model

One-hot (OH) vectors were used to represent amino acid sequences in ${\mathbb{R}}^{d}$ . Each vector describes the AA positions of the HLA of one population. These vectors, $X_{ij}$ , are used as an input to the regression learning models, and the predicted values $y_{i}$ are each allele log frequencies in the appropriate population.


(2) $$y_{i}=Log\left(f_{i}\right),$$

where $f_{i}$ is the frequencies vector of the *i*-th population. Positions with less than 3 AA differing from the majority AA were ignored.

The OH vectors were the input to an SVR learning algorithm, for each population by itself. When the model was trained on A, B and C loci together, we added a OH vector at the end of the input sequence in order to separate the different alleles. When the model was trained on all the populations together, we added a OH vector at the end of the sequence (after the one-hot vector that separates the alleles) in order to differentiate between alleles that came from different populations.

For each training test division, the Spearman correlations were averaged across all ten trials. The SVR (Support Vector Regression - python scikit-learn library (62)) gave the highest correlation on the test set. We optimized each algorithm separately using NNI (61) on an internal validation set. The parameters for the best algorithm, SVR (the linear and non-linear), are presented in **Table 2**.

**Table 2:** SVR models hyperparameters.

	SVR	Linear SVR
**Normalization**	z_score	–
**Kernel**	RBF	Linear
**C**	1	0.01
**Epsilon**	1	2

The $S_{\beta }$ score assigned to each position was calculated as a sum of the absolute value of the SVR coefficients attribute, which assigned a weight to the features.

### 4.5 Definition of HLA positions

The peptide-binding and the Bw4 positions are shown in **Supp. Mat. Table S5**, as defined in (63, 64).

### 4.6 Estimate of amino acid depth

To create the phylogenetic tree, we split our data into two exons (exon 2 - positions 1-90 and exon 3 - positions 91-183), removed the alleles mentioned in **Supp. Mat. Table S3** and built phylogenic trees for each of the exons separately using the PHYLIP package (50). The trees were built using a Maximum Parsimony algorithm for each locus by itself. We added a single gene from another locus to each such tree (A*01:02 for B and C, and B*07:03 for A). We checked that the root is indeed between the outgroup and all the alleles within this locus for all three loci. A Fitch algorithm was then used to estimate the sequence of the internal nodes in the tree.

Then, for each node in the tree, we calculated its level in the tree ( l ) so that the level of the node is set to be the level of its son node plus 1, and the level of the leaves is set to be 0 as follows:


(3) $$l_{leaf}=0\;l_{i}=l_{i-1}+1$$

Node i−1 is the descendant of the node i . Note that all the leaves in the tree are the alleles and the edges are the sequences composed of the amino acids. For each position in the sequence and each level, we calculated the probability of each amino acids at this level in the tree.

To compare the weights vector ( β ) to the phylogenetic results, we defined H as the age matrix as follows:


(4) $$H_{k,j}=\frac{\sum_{i=1}^{l_{k}}i\cdot p_{ij}}{\sum_{i=1}^{l_{k}}p_{ij}},$$

where j is the amino acid, $l_{k}$ is the number of levels on the tree at the k -th position and $p_{ij}$ is the probability of amino acid j in level i . We consider each value in the matrix as the depth of the j -th amino acid at the k -th position.

### 4.7 Correlation between beta and depth

To compare the weights vector, β to the depth above, we compared the two matrices: $H^{20x183}$ representing the age rate of each amino acid in each position, and $\beta^{20x183}$ representing the coefficient of each amino acid on each position (foreach population). We checked the correlation between the beta and the depth values for each locus and each population (as a single flattened vector). We ignored AA absent from the data at any positions.

### 4.8 *DN/DS* based estimates of selection

We used the nucleotide sequence of all the alleles for each of the loci from the (65) site (we ignored alleles containing non AA codes). We separated the sequence into codons, 3 nucleotides in each codon and converted each codon to its corresponding amino acid. For each column (each codon), we calculated the number of mutants in this column, and counted the number of mutants whose amino acid differs from the amino acid of the consensus in that column (*diff_aa_mutants*), where the consensus was based on the most frequent nucleotide in the same position among all loci. Then, for each column, we took the consensus codon, and changed each of its three nucleotides to the three remaining nucleotides. We converted these 3 nucleotides to amino acid and counted the number of amino acids different from the original amino acid (we divided by 9 to get a number between 0 and 1). We multiplied that number by the number of mutants in each codon to get the expected number of different amino acids. In order to get the real number of different amino acids for each codon, we divided the *diff_aa_mutants* value by the number of mutants in that codon.

Finally, for each codon, we calculated the ratio between the expected number of different amino acids and the real number of different amino acids and calculated the Chi-Square value by the following formula:


(5) $$Chi-Square=\frac{{\left(NumRealChangeAA-NumExpectedChangeAA\right)}^{2}}{NumExpectedChangeAA}$$

and extracted the corresponding p-value for each codon.

### 4.9 MHC-I structures

We have analyzed several structures: 6TDQ (66) for visualization of PB and NPB regions, 1AO7 (67), 3WOW (68) and 1BD2 (69) for TCR-MHC-I structures, 1EFX (70), 1IM9 (71) and 5T6Z (72) for KIR-MHC-I structures, and 4NO0 (73), 1P7Q (74) for LILR-MHC-I structures. For each structure, we used (75) for calculating the distances between the MHC positions and the TCR or the KIR positions. We used PyMOL (53) for computing the positions of their side chain, for calculating the electrostatic surface potential (ESP) and for visualization.

### 4.10 Statistical test for selection

To test for the significant deviation of $S_{\beta }$ at a given position in all loci combined from a null model, we compared the $S_{\beta }$ value to the one obtained in the same position (with the same sequence), when the frequencies of each population were scrambled - i.e. the frequency of a given allele was assigned to a different allele over all loci. Significant positions were defined as positions where $S_{\beta }$ is higher than 95% of the $S_{\beta }$ values in the scrambled model. Note that $S_{\beta }$ is defined as the sum over all the absolute values of the coefficients over all populations and all AA at the appropriate position. The null model was computed over 100 Cross Validations (CV).

To test for AA consistently selected at β(AA,Pos) values of all the significant positions in the NPB region, we performed a Chi-Square test between the sum of the $S_{\beta }$ values for each amino acid and the sum of the $S_{\beta }$ values when mixing all these values over 100 random mixings. We computed how often each AA with a p−value<0.05 appears in these cross-validations.

## Data availability statement

The original contributions presented in the study are included in the article/**Supplementary Material**. Further inquiries can be directed to the corresponding author.

## Author contributions

YL supervised the work and wrote a part of the manuscript RL performed the analysis and wrote the manuscript LL performed part of the analysis. All authors contributed to the article and approved the submitted version.
