# Supplementary material for: Bw4 ligand and direct T-cell receptor binding induced selection on HLA A and B alleles
Source: Front Immunol. 2023 Nov 21;14:1236080. doi: 10.3389/fimmu.2023.1236080 (PMC10703150; doi:10.3389/fimmu.2023.1236080)
Supplement: Supplementary file 1 [file DataSheet_1.pdf]

Supplementary Material

Symbol	Race Group	Sample Size	Global population
**AAFA**	African American	1184776	AFA
**AFB**	African	77984	AFA
**CARB**	Black Caribbean	90538	AFA
**SCSEAI**	South Asian	507364	API
**FILII**	Filipino	144044	API
**HAWI**	Hawaiian or other Pacific Islander	36252	API
**JAPI**	Japanese	90340	API
**KORI**	Korean	208560	API
**NCHI**	Chinese	290480	API
**AINDI**	Other Southeast Asian	110040	API
**VIET**	Vietnamese	113748	API
**EURCAU**	European Caucasian	3472992	CAU
**MENAFC**	MidEast/No. Coast of Africa	198752	CAU
**MSWHIS**	Mexican or Chicano	716492	HIS
**SCAHIS**	South/Cntrl Amer. Hisp.	425480	HIS
**CARHIS**	Caribbean Hispanic	332920	HIS
**CARIBI**	Caribbean Indian	42484	NAM
**AMIND**	North American Indian	109796	NAM
**AFA**	African American*	1353298	
**API**	Asian and Pacific Islander*	993464	
**CAU**	Caucasian*	3671744	
**HIS**	Hispanic*	1141972	
**NAM**	Native American Indian*	152280	

Table S1: Population sample sizes. Eighteen detailed race/ethnic sub-populations and five broad race/ethnic populations were studied. Each category was defined based on the registry donor's self-identified race and ethnicity (SIRE). Broad categories are marked in bold and represent the sum of the detailed categories listed above each broad category in the table. Some populations are merged populations, and we mark the merged population associated with each sub-population.

	Event Number	Recomb.	Major parent	Minor parent
**A**	1	A*02:81	A*32:04	A*68:06
	2	A*69:03	Unknown	A*34:13
	3	A*29:13	A*29:48	Unknown
	4	A*80:04	A*24:73	A*11:97
	5	A*03:82	A*30:75	Unknown
	6	A*24:19	A*24:67	A*30:55
	7	A*24:73	A*01:94	Unknown
	8	A*02:50	A*68:35	A*29:48
**B**	1	B*83:01	B*07:78	B*44:99
	2	B*55:14	B*07:78	B*57:13
	3	B*55:56	B*57:13	B*07:64
	4	B*15:96	B*41:08	B*57:32
	5	B*50:17	B*48:17	B*57:51
	6	B*51:62	B*58:64	B*3:63
	7	B*54:38	Unknown	B*81:06
	8	B*38:63	B*39:79	Unknown
	9	B*40:21	B*15:68	B*57:32
	10	B*58:20	Unknown	B*38:26
	11	B*41:46	Unknown	B*42:18
	12	B*56:21	B*13:62	B*08:14
	13	B*40:75	Unknown	B*08:84
	14	B*13:71	B*13:46	Unknown
	15	B*07:78	B*07:22	Unknown
	16	B*35:66	Unknown	B*27:36
**C**	1	C*16:85	C*07:03	Unknown
	2	C*15:25	Unknown	C*02:23
	3	C*04:77	C*18:08	C*14:92
	4	C*03:88	C*01:73	C*17:19
	5	C*05:62	C*02:23	Unknown
	6	C*14:92	C*01:73	C*14:49
	7	C*01:50	C*01:73	C*08:31
	8	C*03:46	Unknown	C*03:68
	9	C*01:73	C*16:85	C*03:68

Table S2: Recombination events were found by RDP4 on all HLA class I nucleotide sequences.

A	B	C
A*02:81	B*83:01	C*16:85
A*69:03	B*55:14	C*15:25
A*29:13	B*55:56	C*04:77
A*80:04	B*15:96	C*03:88
A*03:82	B*50:17	C*05:62
A*24:19	B*51:62	C*14:92
A*24:73	B*54:38	C*01:50
A*02:50	B*38:63	C*03:46
	B*40:21	C*01:73
	B*58:20	
	B*41:46	
	B*56:21	
	B*40:75	
	B*13:71	
	B*07:78	
	B*35:66	
	B*07:13	
	B*67:02	
	B*73:01	
	B*73:02	

Table S3: The alleles that were removed from the analysis.

	**Beta ABC**	**Beta A allele**	**Beta B allele**	**Beta C allele**	**dn/ds A allele**	**dn/ds B allele**	**dn/ds C allele**
**Kruskal test**	0.002	0.001	0.0006	0.11	0.0005	0.01	0.15
**U-test Exon 2 and Exon 3**	0.4	0.38	0.27	0.003	0.36	0.11	0.28
**U-test between PB and NPB**	2.1e-11	6.03e-08	1.43e-12	4.08e-06	0.0004	2.38e-06	0.001
**U-test between Loops and Helices**	0.05	0.01	0.01	0.06	0.03	0.07	0.02
**U-test between Loops and Groove**	0.27	0.27	0.41	0.35	0.36	0.44	0.18
**U-test between Loops and Bw4**	0.0004	7.66e-05	9.91e-05	0.26	0.0001	0.001	0.08
**U-test between Helices and Groove**	0.01	0.03	0.01	0.008	0.02	0.08	0.09
**U-test between Helices and Bw4**	0.01	0.01	0.001	0.46	0.001	0.008	0.34
**U-test between Groove and Bw4**	0.001	0.0005	0.0009	0.29	0.0001	0.001	0.14

Table S4: Kruskal and U-test p-values. The Kruskal test was performed between all the four separated groups and the U-test was applied between any two groups. All the tests were performed on groups with an empty intersection.

	HLA position							
**Loop**	16-19	39-44	49-56	86-90	106-107	128-131	137-140	176-183
**Helices**	57-75	78-79	84-85	141-175	-			
**Groove**	1-15	20-38	45-48	91-105	108-127	132-136	-	
**PB**	7	9	24	45	59	62-63	66-67	69-70
	73-74	76-77	80-81	84	95	97	99	114
	116	118	143	147	150	152	156	158-159
	163	167	171	-				
**Bw4**	76	77	80	81	82	83	-	
**Exon 2**	1-90							
**Exon 3**	91-183							

Table S5: All groups' positions. NPB region positions are set to be all HLA positions that are not in the PB region.

	HLA position					
**Significant NPB**	1	21	22	34	43	52
	60	65	71	75	79	82
	83	107	109	113	126	127
	131	137	138	142	144	145
	151	153	154	161	177	178
	179	180	182	183	-	

Table S6: Significant NPB positions.

Position	Amino acid
159	Y
118	Y
84	Y
59	Y
7	Y
150	A
143	T
171	Y
147	W

Table S7: AA of insignificant PB positions.

Hyperparameter	Search space
Normalization	['min_max', 'z_score', 'without_norm']
Kernel	['linear', 'rbf', 'poly', 'sigmoid', 'precomputed']
# Components	['without_PCA', 100, 200, 300, 400, 500, 600]
C	[0.0001, 0.001, 0.01, 0.1, 1, 10, 100]
Epsilon	[0, 0.0001, 0.001, 0.01, 0.1, 1, 2, 4]

Table S8: Hyperparameters search space of the SVR model.

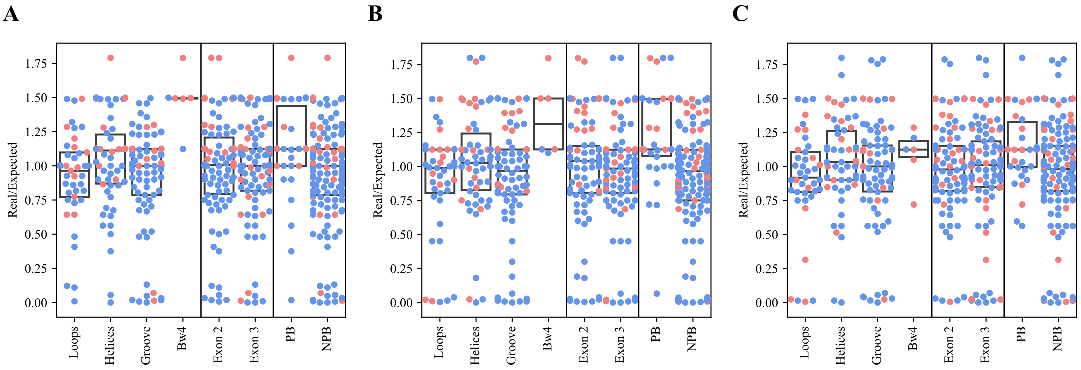

Figure S1: d_n/d_s distribution for A (**A**), B (**B**) and C (**C**) loci separately for each region.

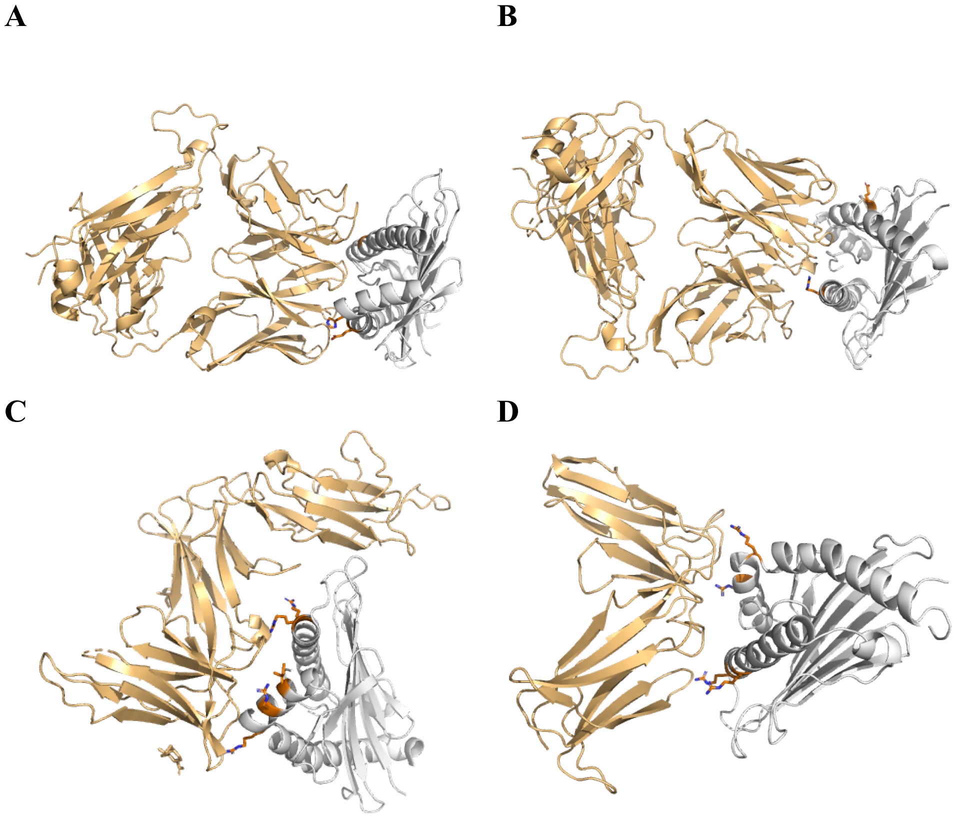

Figure S2: PyMOL visualization of the MHC class I and the positions in the significant NPB region binding to TCR (**A, B**) and KIR molecules (**C, D**). The orange color represents the positions and their side chain.

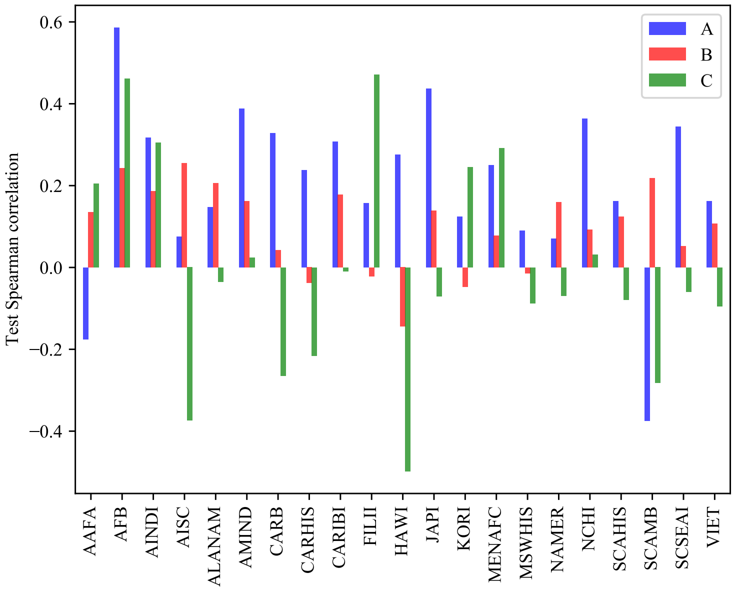

Figure S3: The Spearman correlation between the log real y values (allele's frequencies) and the predicted ones by linear SVR on the test set (the amino acid sequence of each allele) for each locus separately and each population, where the blue bars represent the A locus, the pink bars represent the B locus and the green bars represent the C locus. The model was trained on each of the loci separately.

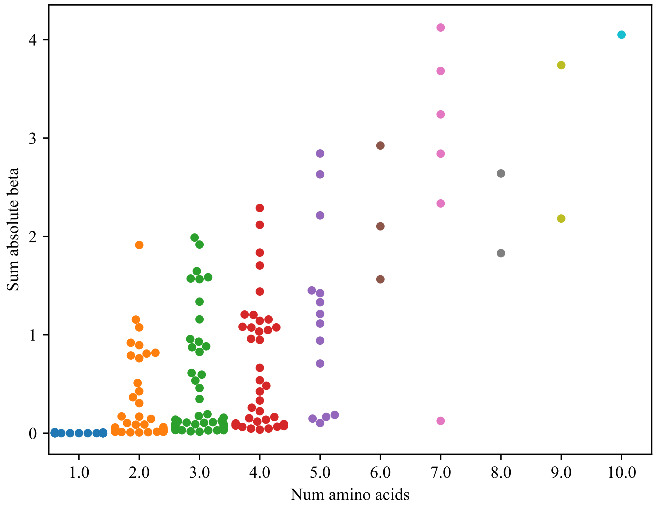

Figure S4: Sum of absolute values of beta vs number of amino acids.

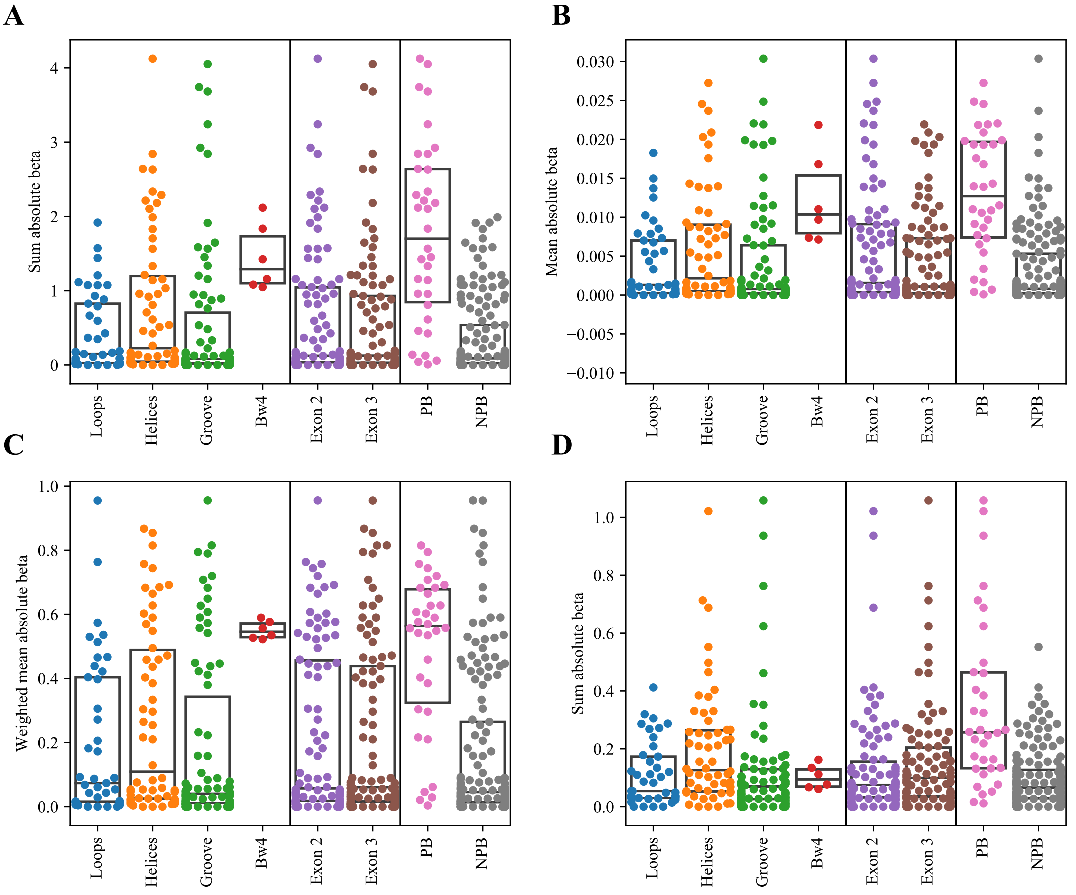

Figure S5: The different methods of beta estimation. **A.** Sum of absolute values of beta. **B.** Average absolute values of beta. **C.** Weighted average of absolute values of beta. **D.** The sum of the absolute values of beta when the model training is performed on all populations together.

	**A locus**	**B locus**	**C locus**	**Mean**
**Model 1**	0.203	0.1	-0.005	0.099
**Model 2**	0.178	0.102	0.032	0.104
**Model 3**	0.373	0.392	0.345	0.37
**Model 4**	0.093	0.044	-0.114	0.007

Table S9: Models accuracy for each locus averaged across all populations using a 10-fold cross-validation. Model 1 was trained separately for each population and each locus. Model 2 was trained separately for each population, considering all loci together. Model 3 was trained using all populations and all loci together. Model 4 was trained similarly to Model 3, with the exclusion of population information from the input for prediction.

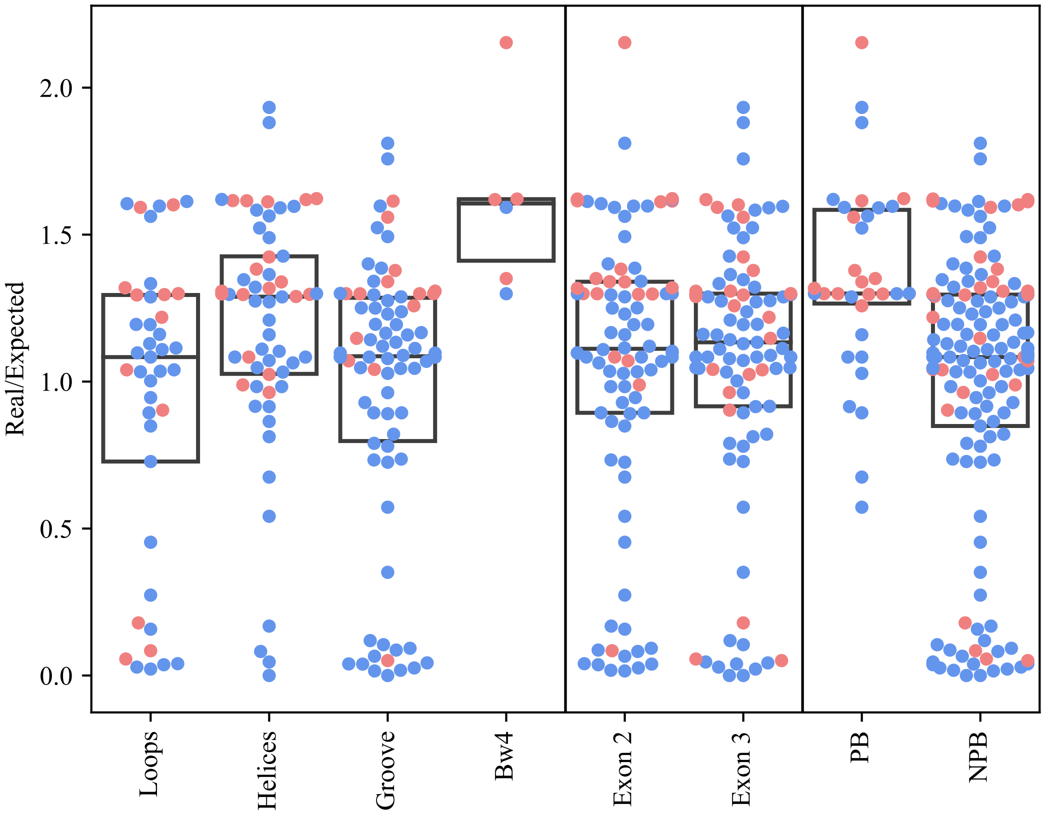

Figure S6: The distribution of the d_n/d_s values for each region. The expected values were calculated according to the Kimura model. Pink dots are significantly different from the null model.

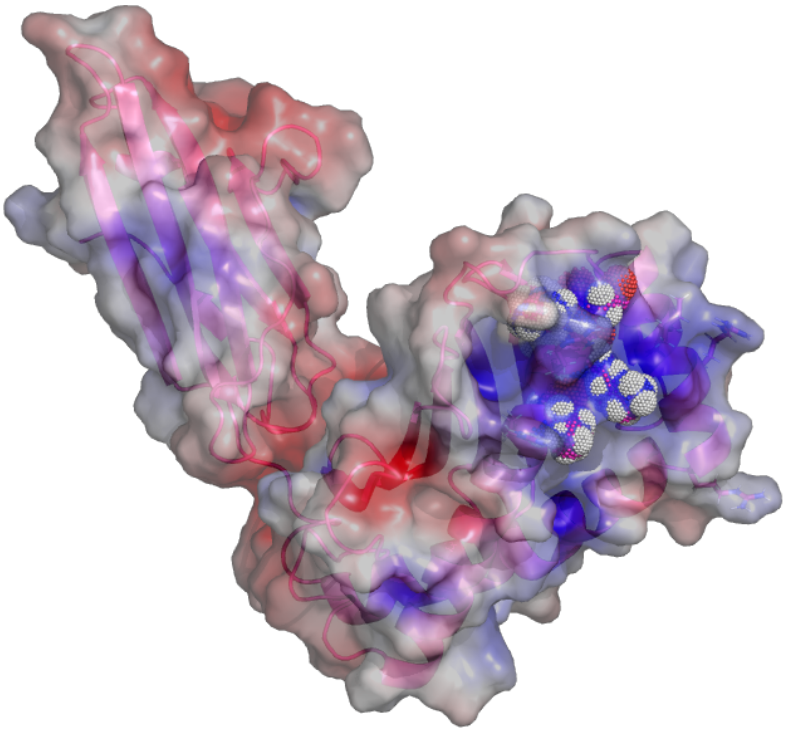

Figure S7: PyMOL visualization for the electrostatic surface potential (ESP) of the MHC. The Bw4 positions are marked as dots. The blue color represents positive EPS and the red color represents negative EPS.

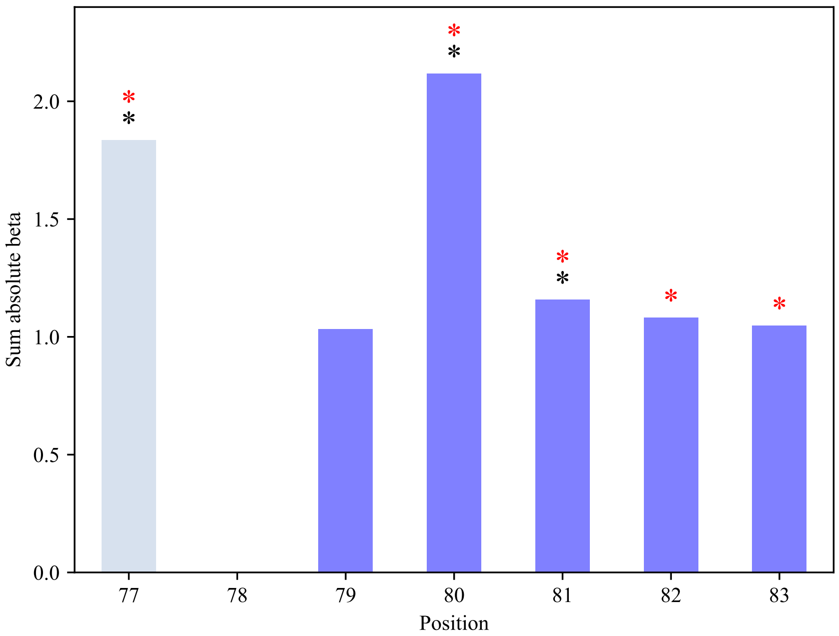

Figure S8: The sum of the absolute of β values for positions 77-83 (the β values are defined as the regression coefficient of the SVR), where the black/red stars represent the peptide-binding or the Bw4 positions respectively, and the dark blue bars represent the significant positions.
